# Selective inhibitor of sodium-calcium exchanger, SEA0400, affects NMDA receptor currents and abolishes their calcium-dependent block by tricyclic antidepressants

**DOI:** 10.3389/fphar.2024.1432718

**Published:** 2024-08-02

**Authors:** Sergei I. Boikov, Tatiana V. Karelina, Dmitry A. Sibarov, Sergei M. Antonov

**Affiliations:** Sechenov Institute of Evolutionary Physiology and Biochemistry of the Russian Academy of Sciences, Saint-Petersburg, Russia

**Keywords:** NMDA receptor, sodium-calcium exchanger, SEA0400, amitriptyline, desipramine, clomipramine, calcium

## Abstract

The open-channel block of *N*-methyl-D-aspartate receptors (NMDARs) and their calcium-dependent desensitization (CDD) represent conventional mechanisms of glutamatergic synapse regulation. In neurotrauma, neurodegeneration, and neuropathic pain the clinical benefits of cure with memantine, ketamine, Mg^2+^, and some tricyclic antidepressants are often attributed to NMDAR open-channel block, while possible involvement of NMDAR CDD in the therapy is not well established. Here the effects of selective high-affinity sodium-calcium exchanger (NCX) isoform 1 inhibitor, SEA0400, on NMDA-activated whole-cell currents and their block by amitriptyline, desipramine and clomipramine recorded by patch-clamp technique in cortical neurons of primary culture were studied. We demonstrated that in the presence of extracellular Ca^2+^, 50 nM SEA0400 caused a reversible decrease of the steady-state amplitude of NMDAR currents, whereas loading neurons with BAPTA or the removal of extracellular Ca^2+^ abolished the effect. The decrease did not exceed 30% of the amplitude and did not depend on membrane voltage. The external Mg^2+^ block and 50 nM SEA0400 inhibition of currents were additive, suggesting their independent modes of action. In the presence of Ca^2+^ SEA0400 speeded up the decay of NMDAR currents to the steady state determined by CDD. The measured IC_50_ value of 27 nM for SEA0400-induced inhibition coincides with that for NCX1. Presumably, SEA0400 effects are induced by an enhancement of NMDAR CDD through the inhibition of Ca^2+^ extrusion by NCX1. SEA0400, in addition, at nanomolar concentrations could interfere with Ca^2+^-dependent effect of tricyclic antidepressants. In the presence of 50 nM SEA0400, the IC_50_s for NMDAR inhibition by amitriptyline and desipramine increased by about 20 folds, as the Ca^2+^-dependent NMDAR inhibition disappeared. This observation highlights NCX1 involvement in amitriptyline and desipramine effects on NMDARs and unmasks competitive relationships between SEA0400 and these antidepressants. Neither amitriptyline nor desipramine could affect NCX3. The open-channel block of NMDARs by these substances was not affected by SEA0400. In agreement, SEA0400 did not change the IC_50_ for clomipramine, which acts as a pure NMDAR open-channel blocker. Thus, NCX seems to represent a promising molecular target to treat neurological disorders, because of the ability to modulate NMDARs by decreasing the open probability through the enhancement of their CDD.

## Introduction

Open-channel block of glutamate ionotropic receptors by endogenous factors, including the external Mg^2+^ block of *N*-methyl-D-aspartate receptor (NMDAR) channels (Nowak et al., 1984; Mayer et al., 1984) and the intracellular polyamine block of α-amino-3-hydroxy-5-methyl-4-isoxazolepropionic acid receptor (AMPAR) channels (Bowie and Mayer, 1995; Koh et al., 1995; for review, Skatchkov et al., 2016), represents one of the most prominent inherent mechanisms of neuronal excitability regulation in the CNS. The same mechanism underlies the therapeutic action of synthetic medicines, such as memantine, amantadine, ketamine, and tricyclic antidepressants (TCA) during treatments of depression, neuropathic pain, neurodegenerative and other neurological disorders (Rogawski and Wenk, 2003) whose pathogenesis is critically determined by NMDAR hyperfunction. Because of the NMDAR contribution to excitatory synaptic transmission, synaptic plasticity, memory formation, brain development, and general functioning, these therapies are often followed by side effects on cognition and behavior (Mimica and Presecki, 2009; for review, Moore et al., 2022).

Some compounds from the above list, which are widely used and demonstrate lesser side effects, memantine (Glasgow et al., 2017), amitriptyline (ATL) (Stepanenko et al., 2019), and desipramine (DES) (Stepanenko et al., 2022), exhibit the open-channel block (Chen et al., 1992; Stepanenko et al., 2019; Stepanenko et al., 2022) combined with the Ca^2+^-dependent inhibition of NMDARs (Stepanenko et al., 2019; 2022). For example, memantine is able to stabilize the Ca^2+^-dependent desensitized state of the GluN1/2A subtype of NMDARs (Glasgow et al., 2017). The removal of Ca^2+^ from the external media abolishes the Ca^2+^-dependent component of the block of NMDAR currents by ATL (Stepanenko et al., 2019) and DES (Stepanenko et al., 2022). The lack of Ca^2+^ causes the compounds to act as pure open-channel blockers, weakening their apparent inhibition and increasing their effective concentrations. Because there is no Ca^2+^-dependent reaction in the NMDAR kinetics of activation other than Ca^2+^-dependent desensitization (CDD), we recently suggested that these agents somehow promote Ca^2+^-dependent desensitization of NMDARs (Stepanenko et al., 2019; Stepanenko et al., 2022).

The process of CDD starts when activated NMDARs allow external Ca^2+^ to enter the cytoplasm. Binding of Ca^2+^ ions to calmodulin forms complexes that interact with the intracellular domain of the GluN1 subunits, followed by a decrease of the probability of the NMDAR open state (Ehlers et al., 1996; for review, Sibarov and Antonov, 2018; Iacobucci and Popescu, 2024). As NMDAR CDD is determined by the near-membrane free intracellular Ca^2+^ concentration ([Ca^2+^]_i_) (Iacobucci and Popescu, 2020), Ca^2+^ exporting mechanisms can be involved in a rapid modulation of either the [Ca^2+^]_i_ and CDD. In particular, inhibition of the sodium-calcium exchangers (NCXs) by its inhibitor KB-R7943 (Sibarov et al., 2015) or by the substitution of Li^+^ for Na^+^ in the external solution (Sibarov et al., 2015) causes a considerable decrease of NMDA-activated currents. Since the removal of Ca^2+^ from the external solution abolishes this effect, the inhibition of NCXs could be attributed as a reason of the indirect influence on NMDARs. On its own, acute NCX inhibition by KB-R7943 (Brustovetsky et al., 2011) does not elevate bulk [Ca^2+^]_i_ in neurons, but KB-R7943 or Li^+^ weaken [Ca^2+^]_i_ clearance (Brustovetsky et al., 2011) that enhances NMDAR CDD. This outcome suggests that during functioning, NCXs permanently extrude incoming Ca^2+^, which otherwise, when disrupted, would promote the NMDAR CDD.

Obviously, there are some phenomenological similarities in the effects of the NMDAR channel blockers (ATL and DES) and inhibitors of NCXs (KB-R7943 and Li^+^) on NMDAR currents. For instance, both types of effects are Ca^2+^-dependent, because the Ca^2+^ removal from the external solution or the cytoplasm of loaded with BAPTA neurons abolishes this dependence (Sibarov et al., 2018; Stepanenko et al., 2019; 2022). Moreover, these influences are prevented by cholesterol extraction from the plasma membranes (Sibarov et al., 2018; Boikov et al., 2022a) and are affected by low ethanol concentrations (Boikov et al., 2020). It should be noted that the Ca^2+^-dependent inhibition of NMDAR currents by ATL and DES could be competitively antagonized by low concentrations of extracellular Li^+^ (Boikov et al., 2022b). Based on these analogies, one may assume that similar to KB-R7943 and Li^+^, ATL and DES, in addition to the open-channel block of NMDARs at the same concentrations, may also inhibit NCXs. This assumption, however, does not seem conclusive because KB-R7943 and Li^+^, as NCX inhibitors, may exhibit NCX-independent effects on NMDARs, pumps, and neurotransmitter transporters, disturbing Ca^2+^ homeostasis (Brustovetsky et al., 2011; Deline et al., 2023).

Earlier, a selective high-affinity NCX inhibitor, SEA0400, with a preferential NCX1 isoform modality of action (Iwamoto et al., 1999; Iwamoto et al., 2004) has been described (Matsuda et al., 2001). In general, this compound represents a good tool to obtain conclusive evidence to prove the inhibition of NCXs by ATL and DES if one could demonstrate a competition between these tricyclic antidepressants and SEA0400. To achieve these goals, here we investigated whether NCX inhibition by SEA0400 at nanomolar concentrations, which do not exhibit any side effects, can influence the currents through native di- and triheteromeric NMDARs composed of GluN1, GluN2A, and GluN2B subunits in rat cortical neurons of primary culture (Zhong et al., 1994; Sheng et al., 1994; Li et al., 1998). Then, we tested the possibility that SEA0400 can interact with the Ca^2+^-dependent inhibition of NMDARs by tricyclic antidepressants, ATL, DES, and clomipramine (CLO).

## Methods

### Animals

All animal procedures were carried out on Wistar rats in accordance with the Federation for Laboratory Animal Science Associations (FELASA) criteria and were approved by the Sechenov Institute’s Animal Care and Use Committees. Rats were kept four to six per cage at an ambient temperature of 22°C–25°C with a 12-h day/night cycle and free access to water and food. Overall, embryos of ten pregnant rats were obtained for the preparation of primary cultures of cortical neurons (about two coverslips with culture per embryo).

### Preparation of primary culture of cortical neurons

The 17-day pregnant rats were provided by the Sechenov Institute Animal Facility. The animals were sacrificed by inhaling CO_2_ for 1 min in a plastic container attached to a CO_2_ tank. Fetal brains were used to make primary cultures of rat cortical neurons, as previously described (Mironova et al., 2007). Neurons were cultured on 7 mm glass coverslips coated with poly-D-lysine in a “Neurobasal” culture medium supplemented with B-27 (Thermo Fisher Scientific, Waltham, MA, United States). Experiments were performed after 10–14 days in culture (Mironova et al., 2007; Han and Stevens, 2009).

### Patch-clamp recording

Whole-cell currents were recorded from cultured rat cortical neurons using a MultiClamp 700B patch-clamp amplifier. Recordings were low-pass filtered at 400 Hz and digitized at the acquisition rate of 20,000 samples per second by Digidata 1440A, controlled by pClamp v10.6 software (Molecular Devices). Solution exchange was performed by means of a fast solution application system, as described earlier (Sibarov et al., 2015). Unless otherwise specified, the external bathing solution contained (in mM): 144 NaCl; 2.8 KCl; 1.0 CaCl_2_; 10 HEPES, at pH 7.2–7.4, osmolarity 310 mOsm. The intrapipette solution contained (in mM): 120 CsF, 10 CsCl, 10 EGTA, and 10 HEPES, osmolarity 300 mOsm, with pH adjusted to 7.4 with CsOH. Patch pipettes of 4–6 MΩ were pulled from RWD B-15086-10F borosilicate capillaries with filament. Experiments were performed at 25°C–28°C. Under control conditions, neurons were voltage clamped at −70 mV. Data are reported without corrections for liquid junction potential, which was measured as −11 mV. To activate NMDARs, 100 µM NMDA was always co-applied with 30 µM glycine as a co-agonist. Some experiments were performed on neurons loaded with BAPTA to bind free intracellular Ca^2+^. With this aim cortical neurons were incubated in a bathing solution containing 5 µM BAPTA-AM for 1 h.

### Reagents

Most compounds were acquired from Sigma-Aldrich, St. Louis, MO, United States. Particularly ATP-Mg cat. A1852; BAPTA-AM cat. A1076; CsCl cat. C4036; CsF cat. 255718; CsOH cat. 232041; EGTA cat. 324626; Glycine cat. G7126; GTP-Na cat. 51120; HEPES cat. 54457; KB-R7943 cat 4144; KCl cat. P3911; NaCl cat. S9888; NMDA cat. M3262; poly-D-lysine cat. P1024. SEA0400 cat 6164/10 was from Tocris Bioscience, Minneapolis, United States. Neurobasal medium cat. 21103049 and B-27 supplement cat. A3582801 were from Thermo Fisher Scientific, Waltham, MA, United States.

### Analysis of membrane currents

To determine the blocking potency of TCAs, NMDA-elicited currents were recorded in the absence and presence of different TCA or SEA concentrations ([B]). Amplitudes of currents measured in the presence of blocker (I_b_) were normalized to the maximal current response in control (I_c_). The IC_50_ and the Hill coefficient (*h*) were estimated in Origin 2021 software (OriginLab) by fitting concentration-inhibition curves with the Hill equation: I_b_/I_c_ = 1 + (*m*-1) * [B]^h^/([B]^h^ + IC_50_^h^), where *m* is a free parameter indicating a fraction of current, which cannot be inhibited by any [SEA0400].

The desensitization kinetics of NMDARs was fit in ClampFit (pClamp, Axon Instruments) using the single exponential function.

### Statistical analysis

The data are presented as representative measurements as well as mean values ±standard error of the mean (SEM). The sample number (n) refers to the number of recorded cells. Data pairs were compared using Student’s two-tailed t-test. Multiple groups were compared using one-way ANOVA with post-hoc Tukey’s test. Statistical significance is reported in the figures according to the following symbols: *, *** and ****, which indicate *p* values below (<) 0.05, 0.001, and 0.0001 respectively. Curve fitting was performed using OriginPro software (OriginLab Corp.). IC_50_s obtained from individual experiments performed in the same experimental conditions were averaged to get mean ± SEM values.

## Results

### SEA0400 affects NMDAR currents

**FIGURE 1:**
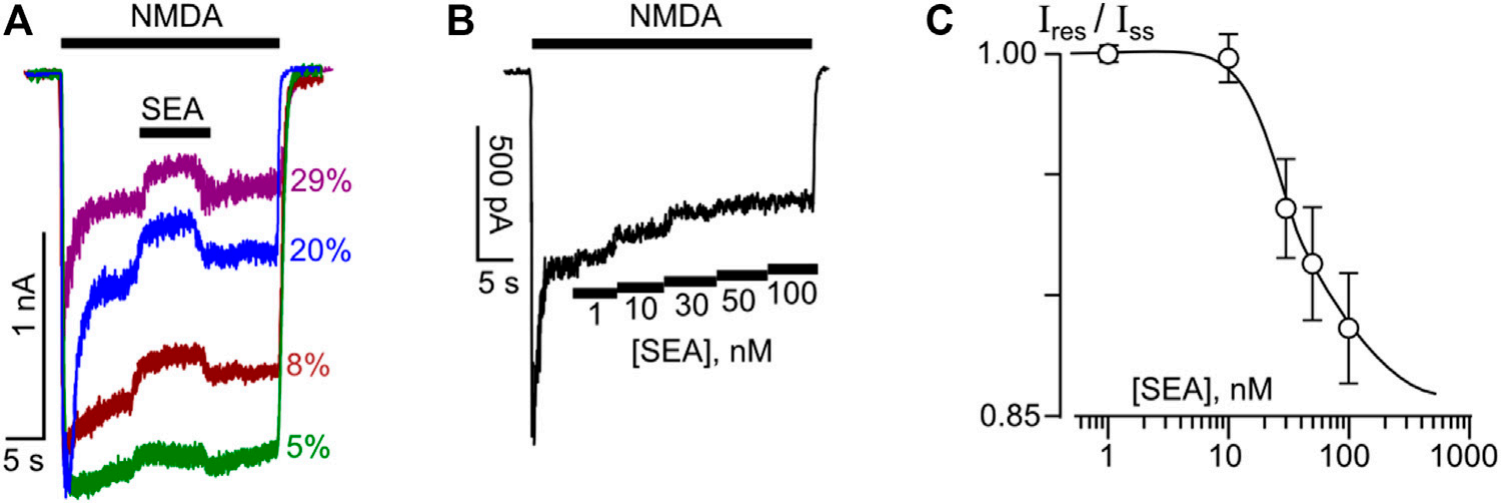
Inhibition of NMDAR currents by SEA0400 in cortical neurons. **(A)** An overlay of NMDAR currents elicited by 100 μM NMDA +30 μM Gly in 4 neurons recorded at −70 mV in the presence of 1 mM [Ca^2+^]. SEA0400 (50 nM) was applied at the steady state of NMDA-activated currents. Percentages (%) exhibit fractions of inhibited currents. **(B)** Sample trace of NMDAR currents recorded in the bathing solution containing 2 mM [Ca^2+^] at −70 mV in the presence of increasing SEA0400 concentrations as indicated by numbers below the trace in nM ([SEA]). **(C)** An average concentration-inhibition relationship for SEA0400 effect on NMDAR currents in the bathing solution containing 2 mM [Ca^2+^] obtained from 8 experiments. Symbols show mean values ± S.E.M of the relative amplitudes (I_res_/I_ss_) of currents recorded in the presence (I_res_) and absence (I_ss_) of different SEA0400 concentrations [SEA]. Solid line is the fit to the data with the Hill equation, that yielded the IC_50_ value of 27 ± 4.9 nM (n = 8).

We have started from the investigation whether low nanomolar concentrations (within the range from 1 nM to 100 nM) of a selective NCX1 inhibitor, SEA0400, may influence NMDA-activated currents of cortical neurons. With this aim, the effects of 50 nM SEA0400, which was applied at the steady state of currents evoked by 100 μM NMDA (+30 μM Gly as a co-agonist) in neurons, were studied. In the external bathing solution (contained 1 mM Ca^2+^), SEA0400 caused a moderate but reliable decrease of the steady-state amplitudes of NMDAR mediated currents. The degree of inhibition noticeably varied between neurons (Figure 1A).

To obtain the concentration-inhibition relationships for the SEA0400 effect on NMDARs, SEA0400 concentrations ([SEA]) from 1 nM to 100 nM were applied to the steady state of NMDA-activated currents by a stepwise increase of concentration (Figure 1B) in the external bathing solution with 2 mM Ca^2+^. The increase of [SEA] induced a progressive decrease of the amplitudes of currents. The amplitude values were plotted as a function of [SEA], and the IC_50_ value for SEA0400 inhibition of NMDA-activated currents of about 27 nM was measured by fitting the data with the Hill equation (Figure 1C). This value matches well the IC_50_ value of 33 nM for SEA0400 inhibition of NCX in neurons reported earlier (Matsuda et al., 2001).

**FIGURE 2:**
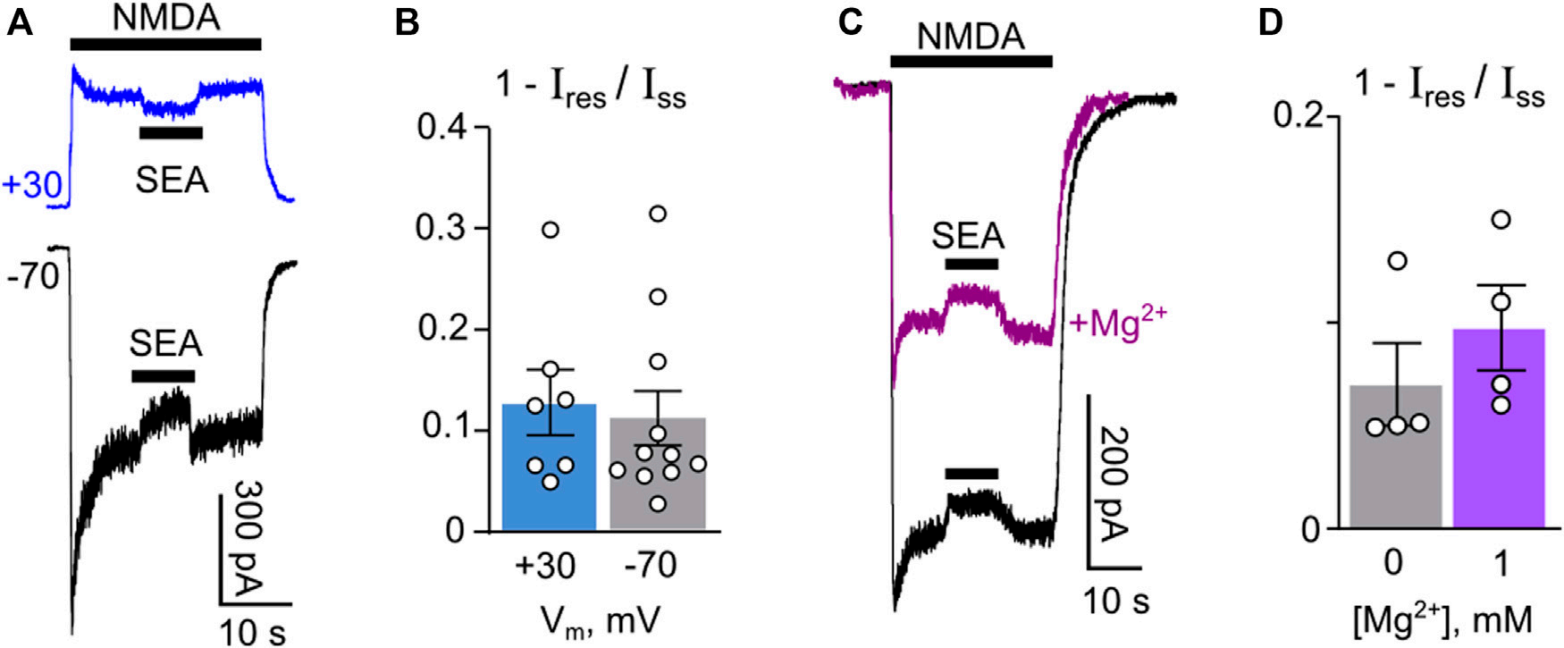
Challenge of the open-channel block as the mechanism of SEA0400 effects on NMDAR currents. **(A)** Sample traces of NMDAR currents recorded in a single neuron at −70 mV and +30 mV in the bathing solution containing 1 mM [Ca^2+^], 50 nM SEA0400 (SEA) was applied to the steady state of NMDA-activated currents. **(B)** The histogram represents the fractions of SEA0400 blocked current at two membrane voltages (1 − I_res_/I_ss_, where I_res_ is the amplitude of residual current recorded in the presence of SEA0400 and I_ss_ is the amplitude of the steady-state current in control conditions) obtained in each of experiments (circles). **(C)** Sample traces of NMDAR currents recorded in a single neuron at −40 mV in the bathing solution containing 1 mM [Ca^2+^] in the absence and in the presence of 1 mM [Mg^2+^]. 50 nM SEA0400 was applied to the steady state of NMDA-activated currents. **(D)** The histogram represents the fractions of SEA0400 blocked current in the absence and in the presence of Mg^2+^ (1 − I_res_/I_ss_, where I_res_ is the amplitude of residual current recorded in the presence of SEA0400 and I_ss_ is the amplitude of the steady-state current in control conditions) obtained in each of experiments (circles).

Although it is unlikely that this SEA0400 concentration may induce an open-channel block of NMDARs, to exclude this mechanism from consideration we further studied the possible dependence of the SEA0400 effect on membrane voltage, which presents an inherent property of open-channel block for compounds exhibiting rather fast kinetics as observed for SEA0400. In experiments on NMDAR currents recorded at −70 mV and +30 mV, 50 nM SEA0400 induced a similar decrease of the steady-state amplitude (Figures 2A, B). We further tested whether SEA0400 is able to compete with external Mg^2+^ for NMDAR channels or their effects are independent. In experiments, 1 mM of external Mg^2+^ caused about a half-amplitude decrease of NMDA-activated currents (Figure 2C). Application of 50 nM SEA0400 to the steady state of currents both without and in the presence of Mg^2+^ induced similar decreases of their amplitudes (Figure 2C). While some tendency of an increase of the fraction of SEA0400 inhibited current in the presence of Mg^2+^ was observed, the mean values measured in both conditions did not differ (Figure 2D), suggesting additive effects of Mg^2+^ and SEA0400 rather than competitive. From these experiments, we may conclude that SEA0400 interacts with molecular target other than NMDAR channels.

### Calcium-dependence of SEA0400 effects

**FIGURE 3:**
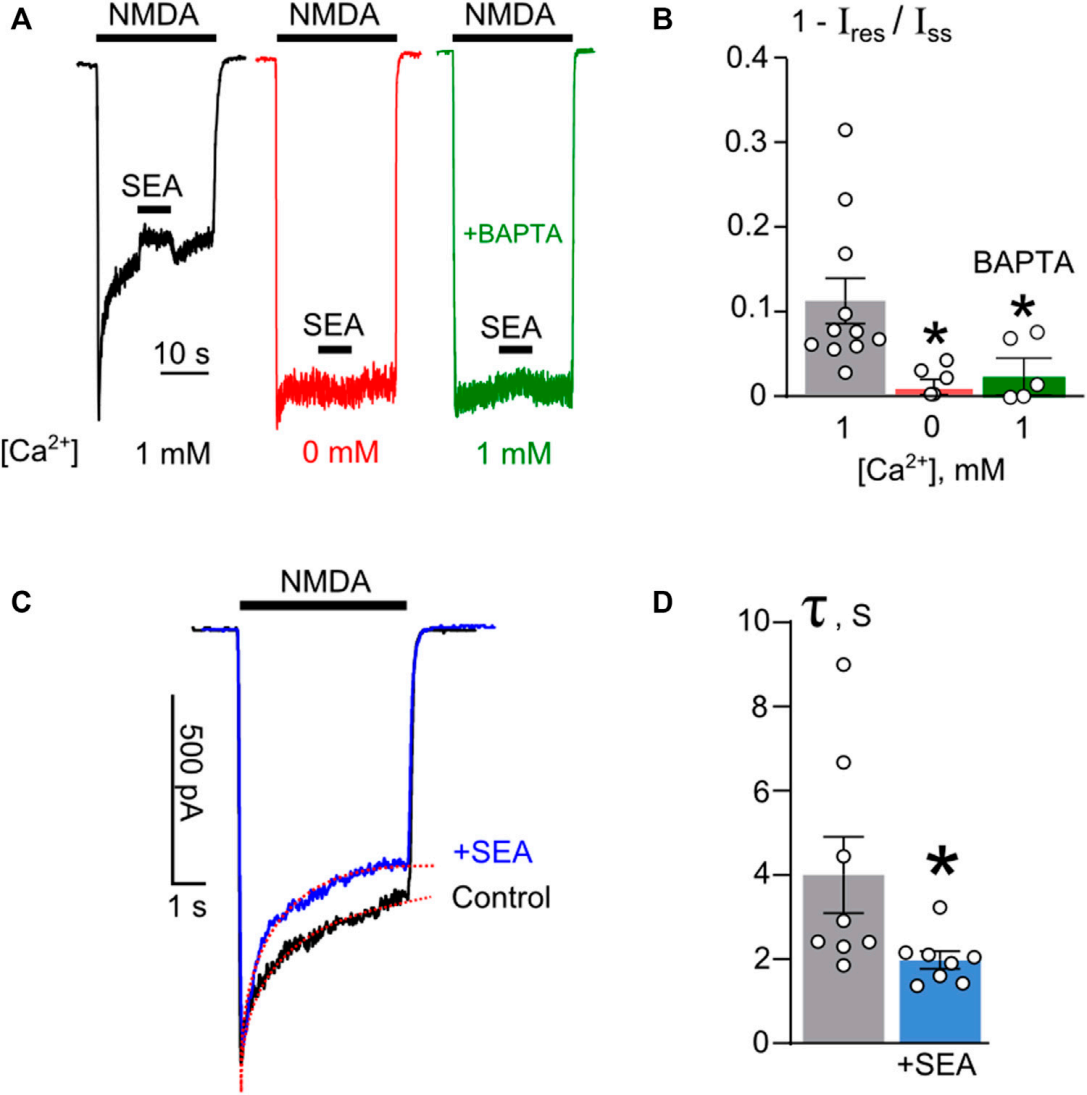
The Ca^2+^-dependence of SEA0400 inhibition of NMDAR currents. **(A)** Currents normalized to their peaks activated by 100 μM NMDA +30 μM Gly recorded at −70 mV in the bathing solution containing 1 mM [Ca^2+^] (black), in Ca^2+^-free solution (red), and in the presence of 1 mM [Ca^2+^] in BAPTA-loaded neuron (green). NMDA and 50 nM SEA0400 applications are indicated by bars above the traces. **(B)** The histogram represents the fractions of SEA0400 blocked current (1 − I_res_/I_ss_, where I_res_ is the amplitude of residual current recorded in the presence of SEA0400 and I_ss_ is the amplitude of the steady-state current in control conditions) obtained in each of experiments (circles). Mean values ±S.E.M. are compared. *- data are significantly different (*p* = 0.03, n = 5, one-way ANOVA with post-hoc Tukey’s test). **(C)** An overlay of currents activated by 100 μM NMDA +30 μM Gly recorded at −70 mV in the same cell in control (black) or in the presence of 50 nM SEA0400 (+SEA, blue) in the bathing solution containing 1 mM [Ca^2+^]. Red lines are single-exponential fits of decays of currents. **(D)** In histogram decay time constant (τ) values of amplitude declines from the peak to the steady state of currents in control and in the presence of 50 nM SEA0500 (+SEA), as indicated on panel C, are compared. Circles depict τ values obtained in each of experiments. Mean values ±S.E.M are indicated by columns and error bars. * - data are significantly different (*p* = 0.02, n = 8, paired Student’s two-tailed t-test).

Whereas the open-channel block does not contribute to the influence of SEA0400, the effect, however, exhibited remarkable dependence on extracellular Ca^2+^. When Ca^2+^ was omitted from the external bathing solution, this compound did not cause any decrease of the steady-state amplitude of NMDA-activated currents (Figure 3A). This observation raises a question of whether Ca^2+^ acts from the outside or Ca^2+^ influx through open channels of activated NMDARs contributes to SEA0400 effects from the inside of neurons. To clarify this issue, SEA0400 inhibition of NMDAR currents was examined in the presence of 1 mM external Ca^2+^ on neurons loaded with a Ca^2+^ chelator, BAPTA. Under these particular conditions, neurons did not exhibit desensitization as well as SEA0400 did not cause NMDAR inhibition (Figures 3A, B). Therefore, intracellular rather than extracellular Ca^2+^ is required for SEA0400 effects.

Given that SEA0400 inhibits NMDAR currents only in the presence of Ca^2+^, we can speculate that SEA0400 affects NMDAR CDD. Most probably, upon inhibition of NCX1, SEA0400 indirectly enhances NMDAR CDD by increasing [Ca^2+^]_i_. To verify this assumption, the rate of NMDAR CDD onset, estimated as the decay time constant of NMDAR current from the peak to the steady state, was analyzed in the absence and presence of SEA0400 (Figure 3C). The decay of NMDA-activated currents recorded in the presence of 50 nM SEA0400 revealed a 2-fold decrease of the time constant value from 4.0 ± 0.9 s measured under control conditions to 2.0 ± 0.2 s that, presumably reflects an acceleration of NMDAR CDD in the presence of SEA0400 (Figure 3D). The acceleration of the CDD onset resulted in a smaller steady-state amplitude of NMDAR currents, than those obtained under control conditions (Figure 3C).

Therefore, in cortical neurons, NCX1 inhibition by SEA0400 reinforces NMDAR CDD, which phenomenologically resembles the effects of open-channel blockers but is voltage-independent and require extracellular Ca^2+^ to enter the cytoplasm upon NMDAR activation.

### SEA0400 prevents Ca^2+^-dependent TCA inhibition of NMDAR currents

The observation that SEA0400 may enhance NMDAR CDD forced us to further study the effects of SEA0400 on the inhibition of NMDAR currents by three TCAs: ATL, DES, and CLO, which vary with the extent of the Ca^2+^-dependence of their effects on NMDARs (Stepanenko et al., 2019; 2022).

**FIGURE 4:**
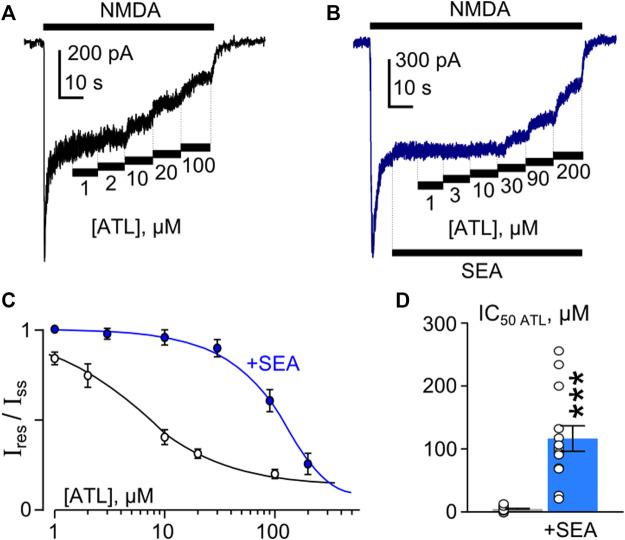
SEA0400 affects amitriptyline (ATL) inhibition of NMDAR currents. **(A,B)** Examples of currents activated by 100 μM NMDA +30 μM Gly recorded at −70 mV in the presence of increasing [ATL] in control **(A)** and in the presence of 50 nM SEA0400 **(B)** in the bathing solution containing 2 mM [Ca^2+^]. **(C)** Average concentration-inhibition relationships for SEA0400 effect on NMDAR currents in control (black) and in the presence of 50 nM SEA0400 (+SEA, blue). Symbols show mean values ±S.E.M of the relative amplitudes of currents (I_res_/I_ss_) in the presence (I_res_) and absence (I_ss_) of different ATL concentrations ([ATL]). Solid lines are the fits to the data with the Hill equation, that yielded the IC_50_ values (Table 1). **(D)** In histogram the IC_50_ values for inhibition of NMDAR currents by ATL in control and in the presence of 50 nM SEA0400 (+SEA) obtained from experiments illustrated in panel **(C)** are compared. Data from each experiment (symbols) and mean values ±S.E.M. are shown. Mean IC_50_ values are summarized in Table 1.

To investigate the concentration-inhibition relationships for the ATL effect on NMDARs, ATL concentrations ([ATL]) from 1 μM to 100 µM were applied by a stepwise increase of concentration to the steady state of NMDA-activated currents in the presence of 2 mM Ca^2+^ (Figure 4A). The increase of [ATL] induced a progressive decrease of the amplitudes of currents. The amplitude values were plotted as a function of [ATL], and the IC_50_ values for ATL inhibition of NMDA-activated currents were measured by fitting the data with the Hill equation. The ATL IC_50_ obtained under control conditions coincides well with the previous data (Stepanenko et al., 2019). In the presence of 50 nM SEA0400 (Figure 4B), however, the concentration-inhibition relationships shifted toward a larger [ATL] (Figure 4C), so that a 23-fold increase of the ATL IC_50_ value from about 5 µM obtained under the control condition to about 116 µM in the presence of SEA0400 was observed (Figure 4D; Table 1).

**TABLE 1:** The IC_50_ values for amitriptyline (ATL), desipramine (DES), and clomipramine (CLO) inhibition of NMDA-evoked currents in cortical neurons.

	ATL (µM)	DES (µM)		CLO (µM)
	[Ca^2+^] = 2 mM	[Ca^2+^] = 1 mM	[Ca^2+^] = 2 mM	[Ca^2+^] = 1 mM
Control	5.0 ± 1.0 (n = 9)	1.7 ± 0.17 (n = 15)	2.2 ± 0.37 (n = 11)	62 ± 6.7 (n = 9)
SEA0400	116 ± 20 (n = 13) ***, *p* = 0.0002	30 ± 4.8 (n = 16) ****, *p* = 0.00001	34 ± 7.9 (n = 10) ***, *p* = 0.0004	67 ± 7.8 (n = 7) ns, *p* = 0.64

*** and **** - the data are significantly different from values obtained in control.

**FIGURE 5:**
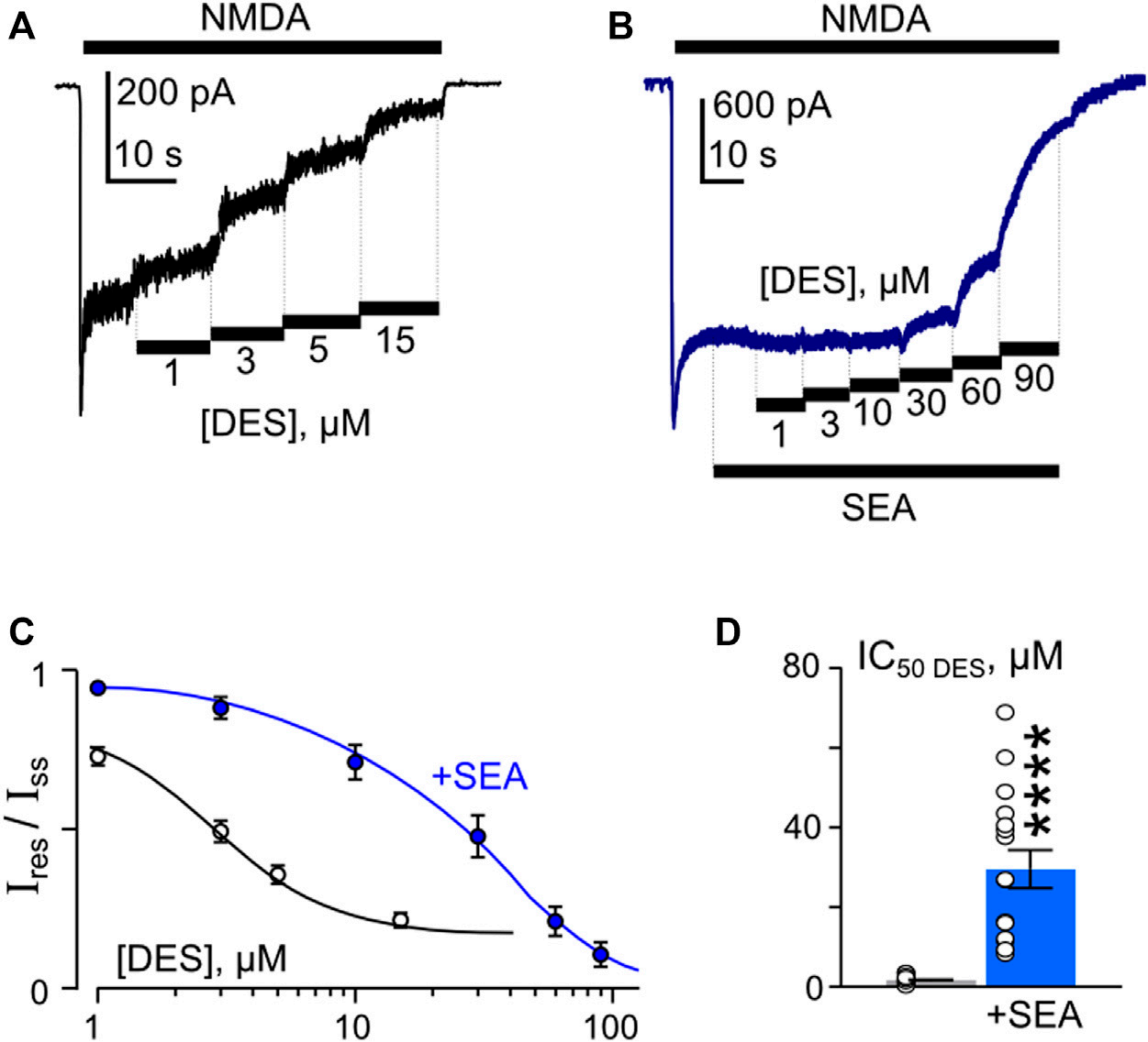
SEA0400 affects desipramine (DES) inhibition of NMDAR currents. **(A,B)** Examples of currents activated by 100 μM NMDA +30 μM Gly recorded at −70 mV in the presence of increasing [DES] in control **(A)** and in the presence of 50 nM SEA0400 **(B)** in the bathing solution containing 1 mM [Ca^2+^]. **(C)** Average concentration-inhibition relationships for SEA0400 effect of NMDAR currents in control (black) and in the presence of 50 nM SEA0400 (+SEA, blue). Symbols show mean values ±S.E.M of the relative amplitudes of currents (I_res_/I_ss_) in the presence (I_res_) and absence (I_ss_) of different DES concentrations ([DES]). Solid lines are the fits to the data with the Hill equation, that yielded the IC_50_ values (Table 1). **(D)** In histogram the IC_50_ values for inhibition of NMDAR currents by DES in control and the presence of 50 nM SEA0400 (+SEA) obtained from experiments illustrated in panel **(C)** are compared. Data from each experiment (symbols) and mean values ±S.E.M. are shown. Mean IC_50_ values are summarized in Table 1.

In similar experiments, the effects of DES within a wide range of [DES] were studied (Figures 5A, B). In 1 mM external [Ca^2+^], the DES IC_50_ values of 1.7 µM and 30 µM were obtained under control conditions and in the presence of 50 nM SEA0400, correspondently (Figures 5C, D). Increasing extracellular Ca^2+^ to 2 mM resulted in quite similar DES IC_50_ values, which were 2.2 µM in control and 34 µM in the presence of SEA0400 (Table 1). The results suggest that SEA0400 induces approximately a 16-fold increase of DES IC_50_ value.

**FIGURE 6:**
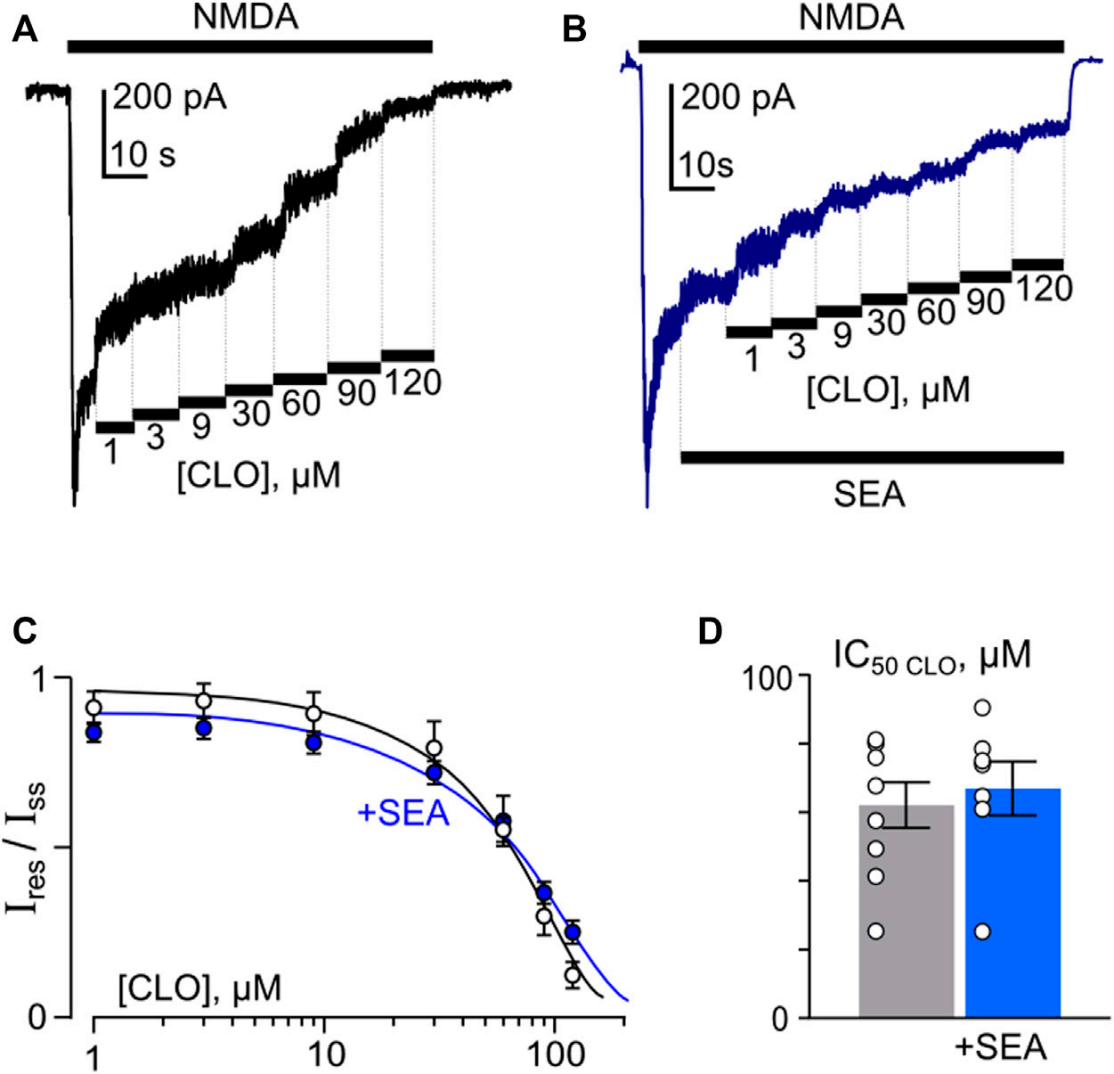
SEA0400 does not affect clomipramine (CLO) inhibition of NMDAR currents. **(A,B)** Examples of currents activated by 100 μM NMDA +30 μM Gly recorded at −70 mV in the presence of increasing [CLO] in control and in the presence of 50 nM SEA0400 in the bathing solution containing 1 mM [Ca^2+^]. **(C)** Average concentration-inhibition relationships for SEA0400 effect on NMDAR currents in control (black) and in the presence of 50 nM SEA0400 (+SEA, blue). Symbols show mean values ±S.E.M of the relative amplitudes of currents (I_res_/I_ss_) in the presence (I_res_) and absence (I_ss_) of different CLO concentrations ([CLO]). Solid lines are the fits to the data with the Hill equation, that yielded the IC_50_ values (Table 1). **(D)** In histogram the IC_50_ values for inhibition of NMDAR currents by CLO in control and the presence of 50 nM SEA0400 obtained from experiments illustrated in panel **(C)** are compared. Data from each experiment (symbols) and mean values ±S.E.M. are shown. Mean IC_50_ values are summarized in Table 1.

In accord to the previous observations (Stepanenko et al., 2019; Stepanenko et al., 2022) ATL and DES exhibit a profound Ca^2+^-dependence of NMDAR inhibition, suggesting a contribution of CDD to their effects, whereas CLO inhibition was Ca^2+^-independent. If SEA0400, somehow, may affect the Ca^2+^-dependent mode of ATL and DES inhibition of NMDARs, then the lack of the SEA0400 effect on the CLO inhibition of NMDARs is expected. In order to clarify this assumption, CLO-induced inhibition of NMDAR currents was studied using the same experimental protocol as for ATL and DES (Figures 6A, B). In agreement with the above consideration, SEA0400 had no effect on CLO IC_50_ of NMDAR inhibition at 1 mM extracellular [Ca^2+^] (Figures 6C, D; Table 1).

Taken together, these observations suggest that there is some interaction between the Ca^2+^-dependent TCA and Ca^2+^-dependent SEA0400 inhibitions of NMDA-activated currents. Because SEA0400 is a high-affinity selective inhibitor of NCX1, this presumably implies competitive relationships between SEA0400 and ATL and/or DES with respect to their Ca^2+^-dependent action on NMDARs. While SEA0400 is present, ATL and DES cannot interact with NCX and perform their own Ca^2+^-dependent inhibition of NMDAR currents. This further supports the conclusion that NCX represents a target for the pharmacological action of ATL and DES as well as NMDARs.

### Mechanisms of SEA0400 interaction with KB-R7943, ATL, and DES

**FIGURE 7:**
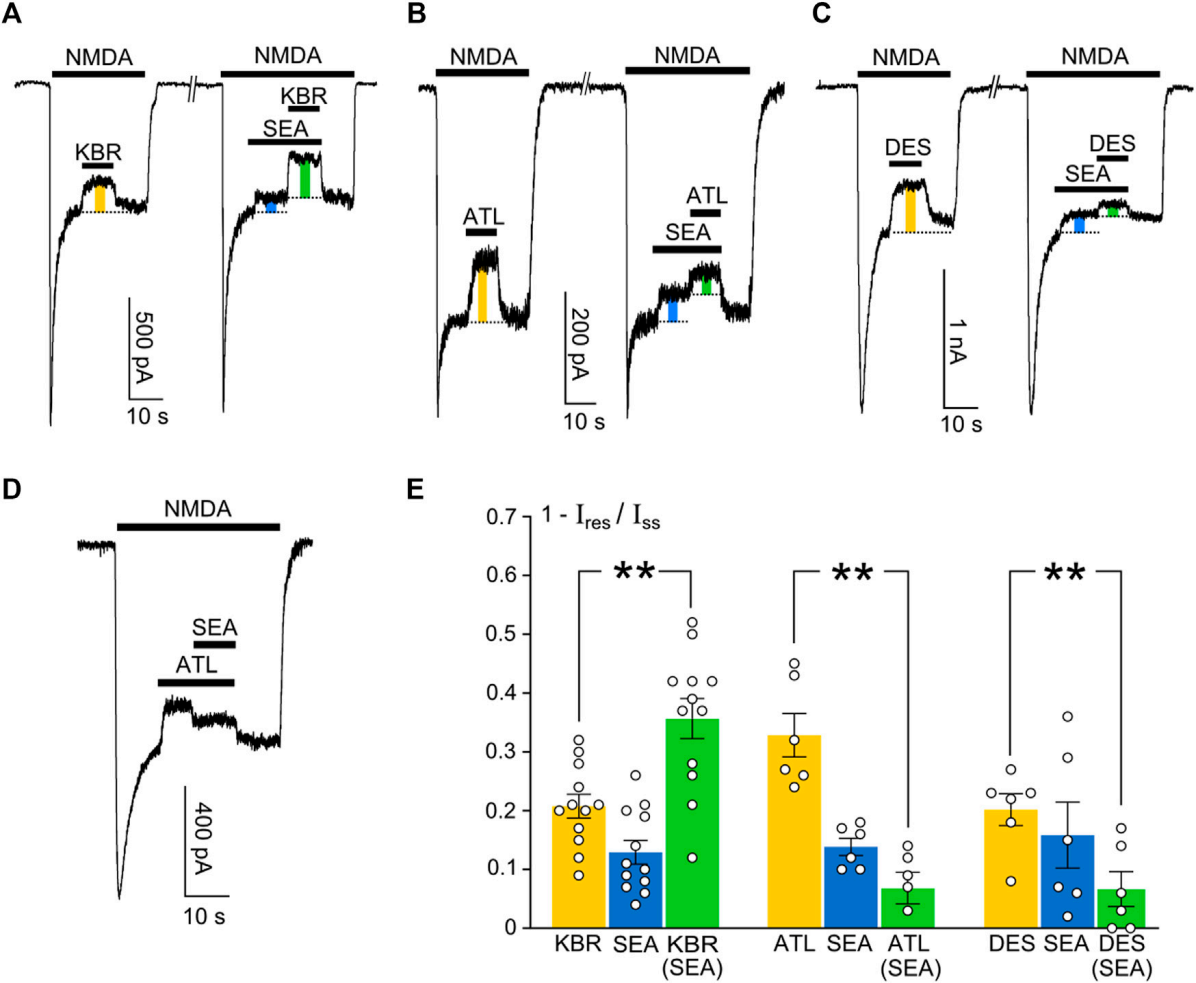
Features of inhibition of NMDAR currents by SEA0400, ATL, DES, and KB-R7943. **(A)** Example of currents activated by 100 μM NMDA +30 μM Gly (NMDA) recorded at −70 mV in the same neuron in the presence of 1 mM Ca^2+^. 10 μM KB-R7943 (KBR), 50 nM SEA0400 (SEA), or KBR in the presence of SEA were applied to the steady state of NMDA-activated currents. Protocol of applications is indicated by bars above the traces. Color bars indicate the fractions of inhibited current. **(B)** Example of currents activated by 100 μM NMDA recorded at −70 mV in the same neuron in the presence of 1 mM Ca^2+^. 10 μM ATL, 50 nM SEA0400 (SEA), or ATL in the presence of SEA were applied to the steady state of NMDA activated currents. Protocol of applications is indicated by bars above the traces. Color bars indicate the fractions of inhibited current. **(C)** Example of currents activated by 100 μM NMDA recorded at −70 mV in the same neuron in the presence of 1 mM Ca^2+^. 1 μM DES, 50 nM SEA0400 (SEA), or DES in the presence of SEA were applied to the steady state of NMDA activated currents. Protocol of applications is indicated by bars above the traces. Color bars indicate the fractions of inhibited current. **(D)** Example of current activated by 100 μM NMDA recorded at −70 mV in the presence of 1 mM Ca^2+^. 10 μM ATL, or ATL in combination with 50 nM SEA0400 were applied to the steady state of NMDA-activated currents. Protocol of applications is indicated by bars above the traces. **(E)** The histogram represents mean fractions of current blocked by ATL, DES, SEA, and KBR (1 − I_res_/I_ss_, where I_ss_ and I_res_ are amplitudes of current before and after application of inhibitor, respectively) obtained in each of experiments (circles). The experimental conditions are shown below the bars. Mean values ± S.E.M are indicated by columns and error bars. ** - data are significantly different (*p* = 0.0092, n = 12 for KBR, *p* = 0.0041, n = 6 for ATL, *p* = 0.0022, n = 6 for DES, one-way ANOVA with post-hoc Tukey’s test).

Whereas an investigation of the concentration-inhibition relationships during an interaction between compounds represents a classical pharmacological approach to prove their competition, effects on currents may provide more insights into the mechanisms of their action. With this aim, we compared an inhibition of NMDA-activated currents by 10 μM KB-R7943 (Figure 7A), 10 µM ATL (Figure 7B), and 1 µM DES (Figure 7C) in control and in the presence of 50 nM SEA0400 in the bathing solution containing 1 mM Ca^2+^. Experiments also were performed in which SEA0400 was applied on the top of the ATL effect (Figure 7D).

KB-R7943 and SEA0400 applied to the steady state of currents caused their inhibition (Figure 7A). In the presence of SEA0400 (a specific NCX1 inhibitor) the fraction of current blocked by KB-R7943 (a preferential NCX3 inhibitor) increased. Such a manifestation exhibits additive or even synergic effects of the compounds on NMDAR currents (Figure 7E), suggesting that both NCX1 and NCX3 are involved in NMDAR CDD modulation.

In contrast to KB-R7943, the fraction of current inhibited by ATL (Figure 7B) and DES (Figure 7C) decreased in the presence of SEA0400 (Figure 7E). This observation is consistent with the conclusion drawn from the IC_50_ measurements that ATL and DES compete with SEA0400 for the same exchanger isoform NCX1 and can not affect NCX3. Here we mean some functional aspect of the competition that could be presented like both compounds compete to prevent the ion traffic through NCX1. It should be noted that the inhibition of currents induced by SEA0400 is less than the inhibition induced by ATL (Figure 7E). Therefore, when SEA0400 acts on the top of the ATL effect, the replacement of ATL and then the occupation of NCX1s by SEA0400 molecules can cause some recovery from the ATL-induced Ca^2+^-dependent desensitization (Figure 7D). This presumably implies some discrepancy in the mechanisms of SEA0400 and ATL actions on NCX1 so that these molecules are not obliged to interact with the same binding site in the NCX1 structure. In addition, based on these experiments we can conclude that at concentrations of ATL and DES used these TCAs do not block open NMDAR channels, otherwise their inhibition would be additive to SEA0400 effects.

## Discussion

Unlike KB-R7943 (Brustovetsky et al., 2011) and Li^+^, SEA0400 appears to be an appropriate compound to inhibit specifically NCXs without side effects on NMDARs and other important molecular participants of intracellular Ca^2+^ homeostasis (Matsuda et al., 2001). Here we demonstrated that at nanomolar concentrations, SEA0400 rapidly and reversibly decreased the steady-state amplitude of NMDAR currents in the presence of extracellular Ca^2+^ only, and the fraction of blocked current did not depend on membrane voltage, similar to KB-R7943 (Sibarov et al., 2015), an inhibitor of NCX, which demonstrates preferential NCX3 modality of action. This somehow contradicts to the observation that depolarization weakened Ca^2+^ entry through recombinant NMDARs in HEK293 cells (Burnashev et al., 1995), and in turn may weaken CDD at +30 mV as compared to −70 mV. It should be taken into account, however that unlike HEK293 cells, neurons express voltage-gated Ca^2+^ channels, which may contribute to Ca^2+^ accumulation in depolarized cells (Reichling and MacDermott, 1993) and enhance CDD.

Since the voltage dependence is an inherent property of the open-channel block by compounds with fast or intermediate blocking kinetics similar to SEA0400, the lack of voltage dependence is not consistent with the open-channel block of NMDARs. Many widely used blockers including ketamine (MacDonald et al., 1991), tricyclic antidepressants (Sernagor et al., 1989), adamantane derivatives such as memantine, adamantine (Parsons et al., 1996; Blanpied et al., 1997), IEM-compounds (Magazanik et al., 1984; Antonov et al., 1998), etc., belong to this group and are clinically well tolerated NMDAR antagonists. This, however, is not relevant for high-affinity slow blockers like MK-801 (Huettner and Bean, 1988; MacDonald et al., 1991), and phencyclidine (MacDonald et al., 1991) which when occlude very slowly dissociate from NMDAR channels [the rate constants of dissociation for phencyclidine and MK-801 are about 0.03 and 0.003 s^-1^, correspondently (MacDonald et al., 1991)] masking the voltage-dependence. This particular feature limits their usage because both drugs induce psychotic behavior (for review, Adell 2020). In addition, NMDARs containing GluN2A and GluN2B subunits could be considered as an uniform receptor population with respect to the open-channel block, and at large blocker concentration the blockade of currents is predicted to be close to complete, which is not the case for SEA0400. Finally, external Mg^2+^ did not weaken the effect of SEA0400, suggesting the lack of competition between these agents. Overall, the results contradict to the capability of SEA0400 to perform NMDAR open-channel block at concentrations studied here.

**FIGURE 8:**
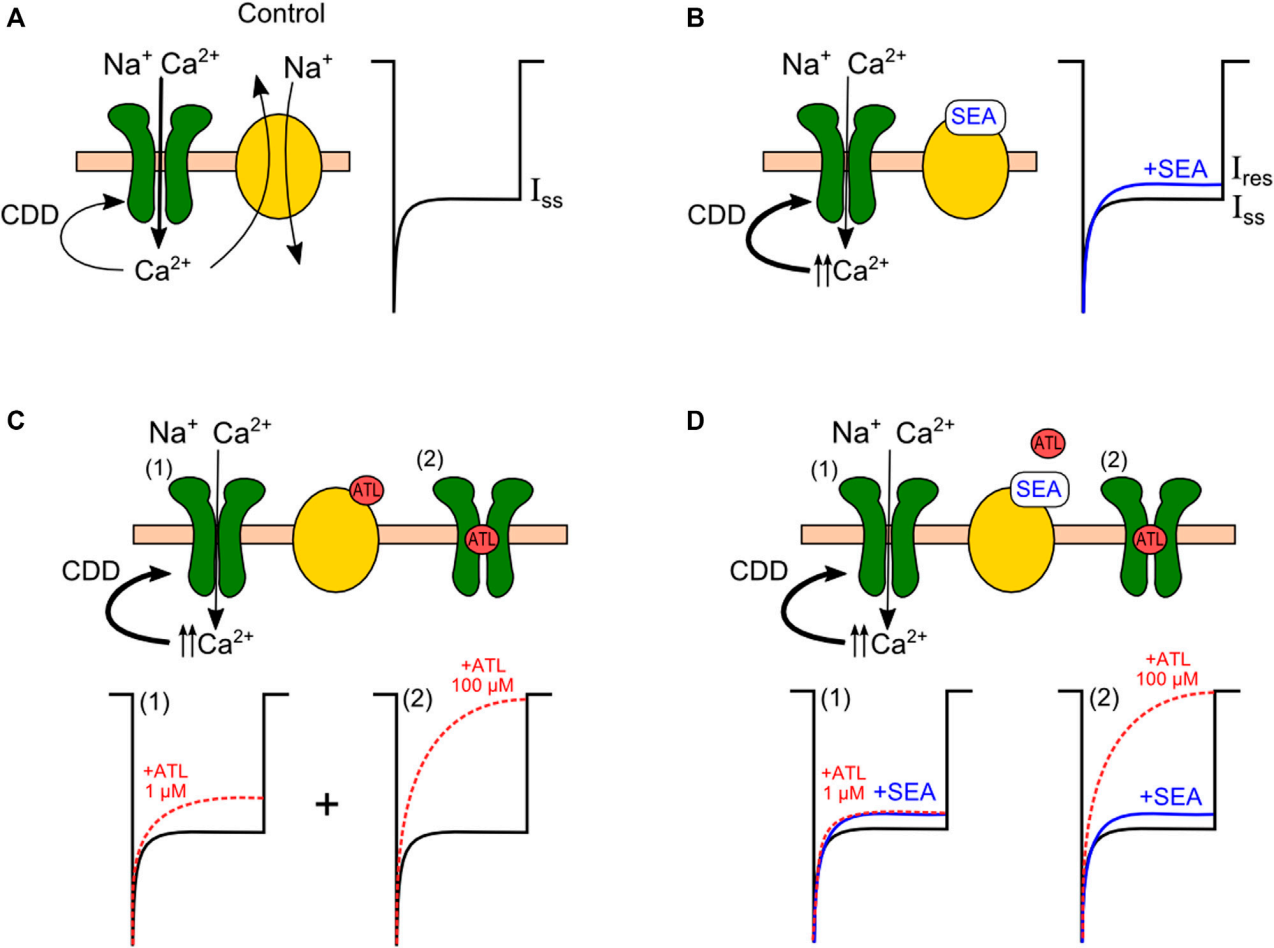
Enhancement of calcium-dependent desensitization (CDD) of NMDA receptors (green) upon inhibition of the sodium-calcium exchanger (yellow). **(A)** Control conditions. The magnitude of the steady-state current of NMDA receptors (I_ss_) is determined by the degree of CDD, which depends on the balance of the Ca^2+^ entry through the receptor and its removal by the exchanger. **(B)** NCX block by SEA0400 leads to intracellular Ca^2+^ accumulation near the receptor, which augments CDD. **(C)** Complex inhibition of NMDAR by ATL. CDD enhancement with low micromolar ATL concentrations (1) is combined with the NMDAR open-channel block by high ATL concentrations (2). **(D)** The Ca^2+^-dependent inhibition of NMDARs by ATL is competitively untagonised in the presence of SEA0400 (1). The pure open-channel block of NMDAR by ATL is observed when NCX is already blocked by SEA0400 (2).

The requirement of intracellular Ca^2+^ highlights the involvement of NMDAR CDD in the SEA0400 effect. This conclusion is further supported by the observation that the decay of NMDAR currents to the steady-state amplitude determined by CDD is accelerated by SEA0400. Since the IC_50_ value for SEA0400-induced inhibition of NMDAR currents matches those for NCX, it is likely, that the compound enhances NMDAR CDD by suppressing NCX extrusion of Ca^2+^. As is illustrated in Figure 8, under these particular conditions, the balance between Ca^2+^ entry through activated NMDARs and its extrusion by NCXs (Figure 8A) most probably is shifted towards intracellular Ca^2+^ accumulation and a consequent enhancement of NMDAR CDD (Figure 8B). Indeed, SEA0400 demonstrated fast kinetics of on- and offsets resembling by these features the direct block of NMDAR channels, but, in contrast, it is determined by the local NCX and NMDAR functional interplay. Furthermore, since SEA0400 is selective to NCX1 the observed manifestation can be attributed to the modulation of NMDAR CDD by this exchanger isoform.

Previously, the potentiation of NMDAR CDD as a mechanism of pharmacological action was demonstrated in experiments with KB-R7943 (Sibarov et al., 2015), Li^+^ (Sibarov et al., 2015), ATL, and DES (Stepanenko et al., 2019; Stepanenko et al., 2022). Even memantine demonstrates this mode of action (Glasgow et al., 2017) that is based on its ability to inhibit NCX (Brittain et al., 2012). However, unlike SEA0400 (Tanaka et al., 2002), these substances are not selective and affect both NCX1 and NCX3 isoforms expressed in neurons (Thurneysen et al., 2002), and in addition, in similar concentrations perform effects on different molecular targets. There are reports that KB-R7943 may inhibit L-type voltage-gated Ca^2+^ channels (Ouardouz et al., 2005), suppresses store-operated Ca^2+^ influx in cultured neurons and astrocytes (Arakawa et al., 2000), blocks open channels of NMDARs (Sobolevsky and Khodorov, 1999; Brustovetsky et al., 2011), and, in parallel, mimics the effects of glycine like a co-agonist of NMDAR glycine binding sites (Sibarov et al., 2015). As for the substitution of Li^+^ for Na^+^ in the external solution, it results in a severe disturbance of glutamatergic synaptic transmission either in pre- and postsynaptic locations, including an increase of spontaneous vesicular and non-vesicular transmitter release and postsynaptic depolarization (Antonov and Magazanik, 1988). The consequences of this procedure become clear in view of the Na^+^-dependence of neurotransmitter transporters, ion pumps, and exchangers whose functions are disrupted in the presence of external Li^+^ (for review, Jakobsson et al., 2017). Therefore, SEA0400 only, being a specific high-affinity NCX1 inhibitor, allowed us here to reveal the pure effect of NCX1 inhibition on NMDAR CDD.

Suggesting a competitive relationship between SEA0400 and the compounds described above (ATL, DES, CLO, KB-R7943, and Li^+^) for the same molecular target, their effects on NMDARs are predicted to be weakened in the presence of SEA0400. We, therefore, further compared the SEA0400 effect on IC_50_s for NMDAR inhibition by ATL and DES. These compounds demonstrate a complex inhibition of NMDARs composed of Ca^2+^-dependent component at low micromolar concentrations and channel block at higher concentrations (Figure 8C). For comparison, we also analyzed SEA0400 effects for CLO which blocks NMDAR channels but does not induce Ca^2+^-dependent NMDAR inhibition at physiological [Ca^2+^]s.

The effect of SEA0400 itself on NMDAR currents was moderate and did not exceed 30% of the full block. It should be noted, that KB-R7943 (10 µM) or the substitution Li^+^ for Na^+^ in the external solution induced a much pronounced decrease of NMDAR currents (by about 60%) (Sibarov et al., 2015). This circumstance probably unveils the involvement of NCX3 as well as NCX1 in the regulation of NMDAR CDD, because both KB-R7943 and Li^+^ are not selective with respect to NCX isoforms (Iwamoto and Shigekawa, 1998; Iwamoto et al., 2004). Surprisingly, in the presence of SEA0400, the IC_50_s for NMDAR inhibition by both ATL and DES raised to high micromolar values, corresponding to direct NMDAR channel block by these substances (Stepanenko et al., 2019; Stepanenko et al., 2022) (Figure 8D). Therefore, in the presence of SEA0400 (50 nM), both ATL and DES completely lost the Ca^2+^-dependent component of their effects. This clearly indicates the competition between SEA0400 and ATL or DES for NCX1-governed modulation of NMDAR CDD. Particularly when NCX is already inhibited by SEA0400, ATL, and DES can not inhibit it further and presumably are unable to increase [Ca^2+^]_i_ and CDD. In contrast, the CLO IC_50_ value for NMDARs was not affected by SEA0400 because, at physiological [Ca^2+^] levels, CLO did not demonstrate the Ca^2+^-dependent component of the NMDAR block.

Neurons predominantly express NCX1 and NCX3 isoforms (Thurneysen et al., 2002). However, at 50 nM, SEA0400 selectively inhibits NCX1 without any side effects on NCX3 or other ion transport proteins, including NMDARs (Tanaka et al., 2002). The complete elimination of Ca^2+^-dependent effects of ATL and DES in the presence of SEA0400 unmasks the role of NCX1 in NMDAR CDD modulation by these substances.

## Conclusion

Under physiological conditions, a mixed channel block of NMDARs by medicines such as memantine and Mg^2+^ ions of liquor is maximally introduced at resting membrane voltages, but depolarization of neurons immediately causes relief from the block. As a result, open-channel NMDAR blockers commonly used to treat neurodegenerative disorders and neuropathic pain should be less effective in depolarized neurons. Conversely, the CDD-dependent inhibition is preserved in depolarized and hyperactive neurons, but weakens at the rest, when [Ca^2+^]_i_ is not elevated. For example, neuropathic pain involves excessive NMDAR function (Zhou et al., 2011), and could be cured by ATL (Bryson and Wilde, 1996), which performs a complex block of NMDARs partially determined by the NCX inhibition (Stepanenko et al., 2019) and KB-R7943 (Huang et al., 2019; Andreeva-Gateva et al., 2024), which is a NCX blocker. Therefore, unlike channel blockers, NCX inhibitors may have additional benefits to prevent the pathological consequences of NMDAR hyperfunction. Thus, NCXs seem to represent a promising molecular target to treat neurological disorders involving excessive NMDAR activity (Zhou and Sheng 2013), because of the ability to modulate NMDARs by decreasing the open probability through the enhancement of their CDD.

## Data Availability

The original contributions presented in the study are included in the article/supplementary material, further inquiries can be directed to the corresponding author.
